# A motion-responsive injectable lubricative hydrogel for efficient Achilles tendon adhesion prevention

**DOI:** 10.1016/j.mtbio.2025.101458

**Published:** 2025-01-04

**Authors:** Shujie Cheng, Jihong Yang, Jianguo Song, Xin Cao, Bowen Zhou, Lan Yang, Chong Li, Yi Wang

**Affiliations:** a Basic Research Key Laboratory of General Surgery for Digital Medicine, Affiliated Hospital of Hebei University, Baoding, 071000, China; b School of Clinic Medicine, Tsinghua University, Beijing, 100084, China; c School of Mechanical and Energy Engineering, Beijing University of Technology, Beijing, 100124, China

**Keywords:** Injectable hydrogel, Lubrication, Motion-responsive, Achilles tendon, Tissue adhesion

## Abstract

Achilles tendon is a motor organ that is prone to tissue adhesion during its repair process after rupture. Therefore, developing motion-responsive and anti-adhesive biomaterials is an important need for the repair of Achilles tendon rupture. Here, we report an injectable lubricative hydrogel (ILH) based on hydration lubrication mechanism, which is also motion-responsive based on sol-gel reversible transmission. The lubrication performance is achieved by zwitterionic polymers as we previously proved, and the sol-gel reversible transmission is enabled by dynamic disulfide bonds. Firstly, ILH was proved to be successfully prepared and lubricated as well as sol-gel reversible via FTIR characterization, rheological measurement and tribological tests. Then, in vitro cell experiments and coagulation tests demonstrated the optimal cytocompatibility and hemocompatibility of ILH. To evaluate the potential of ILH's biofunction *in vivo*, SD rats' Achilles tendon rupture & repair model was established. The animal experiments' results showed that ILH significantly prevented tendon adhesion and thus promote tendon healing by inhibiting TGFβ1-Smad2/3 pathway. We believe this work will open a new horizon for tendon adhesion-free repair.

## Graphical abstract

**Figure:**
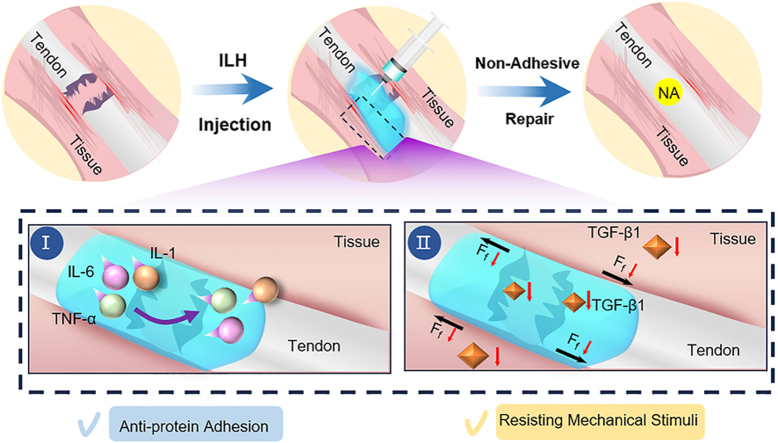

## 1 Introduction

Achilles tendon rupture is a common orthopedic disease, often accompanied with tissue adhesion caused by mechanical activation of fibroblasts, which can lead to local pain and motor dysfunction [[1], [2], [3]]. The large amount of scar tissue adhesion formed by tendon rupture can cause great inconvenience to people who frequently need to move, such as sports athletes [4,5]. Many athletes, due to high-intensity training and frequent competitions, need to use their Achilles tendon to the limit [6]. If an athlete's Achilles tendon is damaged and does not receive precise treatment, it will seriously limit their daily training and have to end their sports career early.

Achilles tendon is wrapped by tendon sheath, which is surface-lubricated and is capable of secreting lubricant, thus ensuring smooth and unobstructed movement during tendon slides [7,8]. When this “lubricating film” is damaged, the lack of protective layer for sports lubrication increases the friction and resistance during tendon movement, leading to enhanced TGF-β1-based mechanical activation of fibroblasts that finally cause severe adhesion [9]. It is a great challenge to protect Achilles tendon from adhesion during its repair process, because the human body's inherent anti-adhesive lubricated tendon sheath cannot self-repair [10]. Therefore, the ideal therapy towards adhesion-free precise regeneration for rupture of Achilles tendon is to develop smart biomaterials that could be highly lubricative at fluid state when tendon moves, while be less lubricative at stable state to protect tissue endogenous healing when tendon rest.

Current clinic therapy for Achilles tendon rupture is to use postoperative anti-adhesion products [11,12], such as Interceed®, DK-film, SprayGel®, CoSeal®. However, these products not only lack lubrication performance, but also cannot be responsive to tendon motion status [13]. In fact, based on clinic experts' usage feedback, these products' anti-adhesion effects are not so satisfied, and some membrane products are not easy to suture in local [14]. According to these problems, in this work we aim to develop a motion-responsive injectable lubricative hydrogel (ILH) based on reversible sol-gel transmission and hydration lubrication theory. Disulfide bond is a covalent bond that is weaker than carbon double bond and possesses reversible bone breaking-forming ability [[15], [16], [17], [18]]. Therefore, disulfide bonds can be used in preparing injectable hydrogels based on its optimal sol-gel reversible performance. Hydration lubrication theory is a classic theory for developing surface super-lubricated materials [19]. It demonstrates that zwitterionic polymer chains could strongly adsorb water molecules through ion-dipole interaction, thus transforming two surfaces' friction into surface verses hydration layer's friction [[20], [21], [22], [23]]. Based on our previous publications, hydration lubricated surfaces are already proved to be able to significantly reduce tissue adhesion *in vivo* [[24], [25], [26], [27], [28]].

**Fig. 1:**
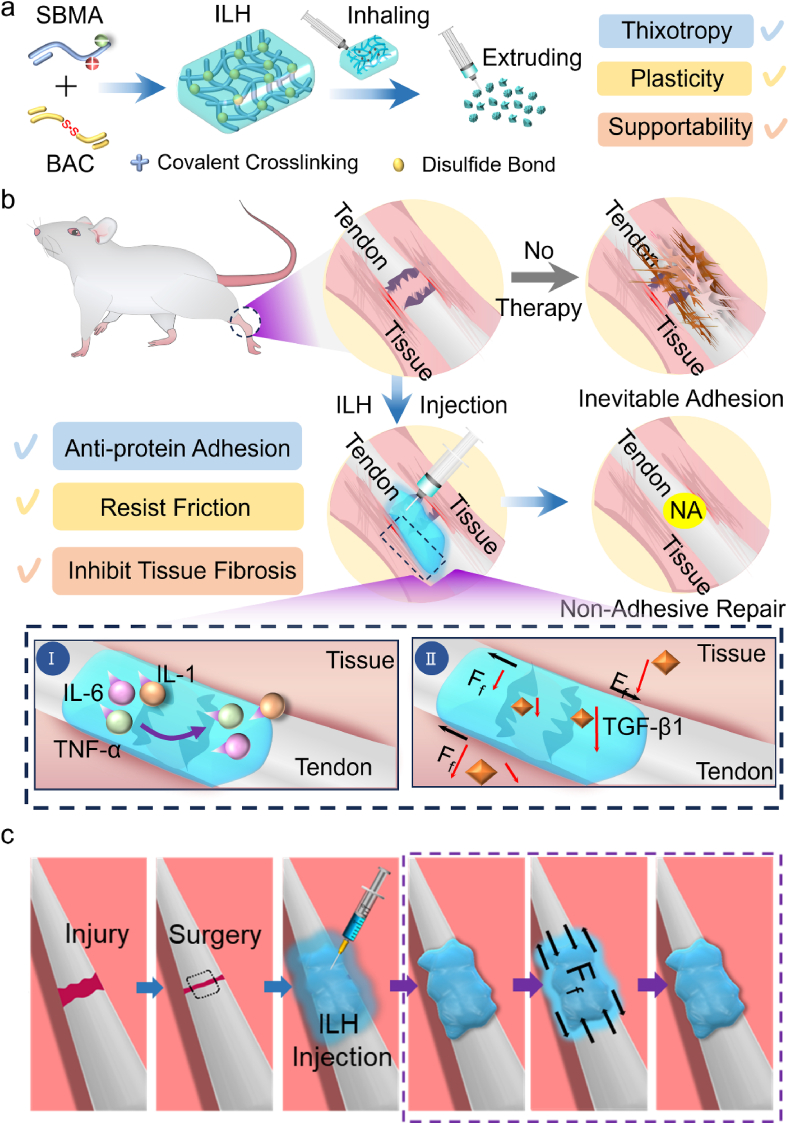
Schematic representation of injectable lubricative hydrogel (ILH) preparation and application. (a) ILH synthesize process; (b) Comparison of no treatment and ILH injection treatment based on a rat tendon rupture model (Schematic diagram of (Ⅰ) inflammatory factors reduction, and (Ⅱ) mechanical stresses resistance). (c) Schematic illustration of the applying process of motion-response ILH *in vivo*.

As shown in Fig. 1a, representative zwitterionic monomer sulfobetaine methacrylate (i.e. SBMA) was free radical copolymerized with Bis(acrylyl)cystamine (i.e. BAC) crosslinker which possesses disulfide bonds, to finally obtain ILH that possessed advantages of thixotropy, plasticity and supportability. As discussed, ILH can resist mechanical stimuli via its motion-responsive lubricating property and further inhibit tissue fibrosis by inhibiting TGF-β1 related pathways (Fig. 1b). Inflammatory factor TGF-β1 is a strong chemokine of fibroblasts that directly promotes the growth of fibroblasts and the formation of collagen. Therefore, the upregulation of TGF-β1 is a sufficient condition for adhesion, and down-regulation of TGF-β1 promotes the repair of ruptured tendons. Fig. 1c illustrates the application process for ILH, where ILH is motion-responsive. ILH is injected into injured tendon surface, and then ILH can promote tendon healing at gel state when tendon rests, meanwhile prevent tendon adhesion at sol gel when tendon moves. In this work, intensive data both in vitro and *in vivo* will be conducted to prove the advantages of ILH as we claimed.

## 2 Experimental section

### 2.1 Material preparation and characterization

**Fig. 2:**
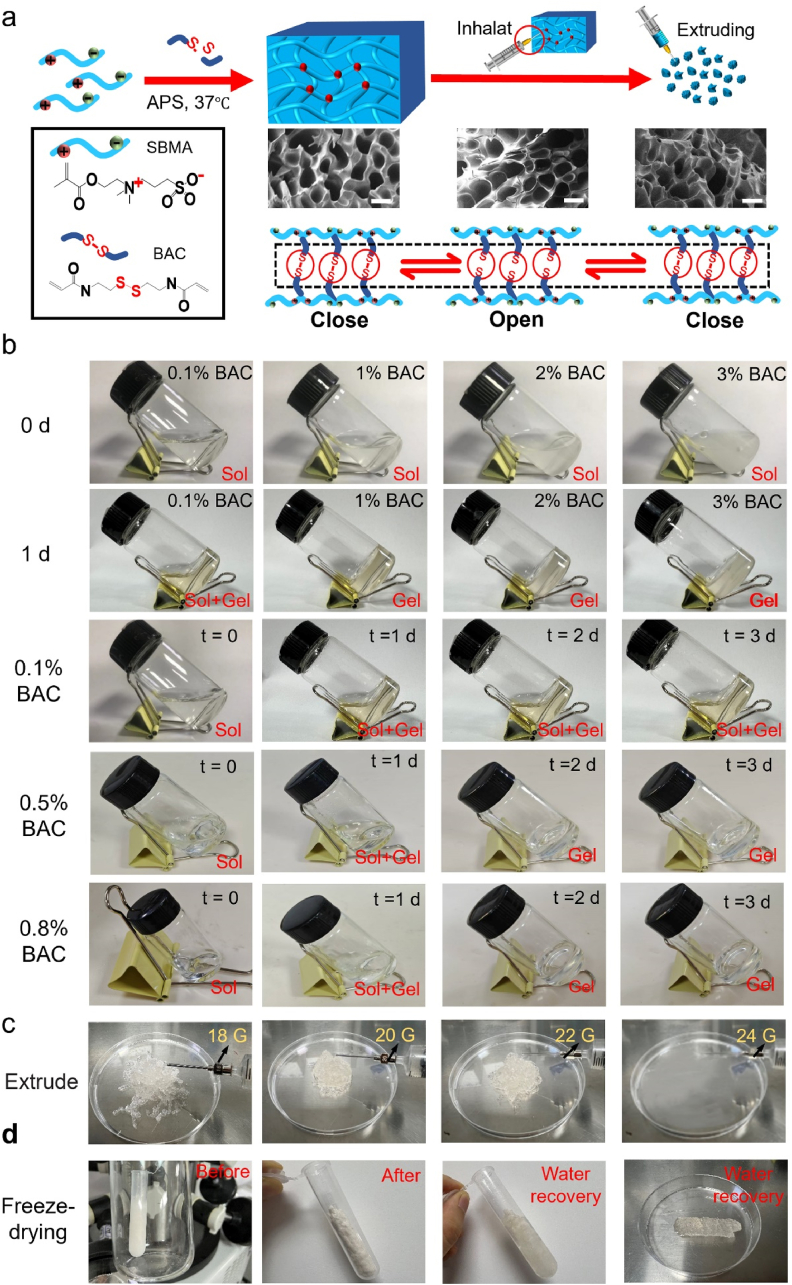
(a) Schematic diagram of ILH preparation and SEM images of ILH structure, scale bar: 20 μm. (b) Photos of the sol-gel transition with different BAC content (0.1 %, 0.5 %, 0.8 %, 1 %, 2 % and 3 %) at different time (37 °C, s: sol, G: gel). (c) Photos of ILH injection using different-sized needles. (d) Photos of ILH storage and use via lyophilization and rehydration processes.

In a reaction vessel filled with nitrogen, as shown in Fig. 2a, crosslinker BAC (0.1 %, 0.5 %, 0.8 %, 1 %, 2 % and 3 % w/w to SBMA) and initiator ammonium persulfate (APS) were added to 40 wt% SBMA aqueous solution for free radical polymerization. The finally reacted solution was purified with a filter with 0.22 μm diameter, dialyzed against water for 24 h, and freeze-dried for storage. The obtained products were ILH.

ILH sample was characterized by SEM (Quanta200, FEI, Eindhoven, Netherlands) for surface morphology analysis from Shiyanjia Lab (www.shiyanjia.com). Samples were pre-sprayed for Pt and then collected for images at least 5 random visions when SEM testing. FTIR (Thermo Scientific Nicolet iS20) was conducted to confirm the specific chemical peaks in ILH chemical structure. Rheometer (Anton paar Inc., MCR 302) from Shiyanjia Lab (www.shiyanjia.com) was used to test ILH's rheological property under oscillation mode at room temperature. The in vitro friction tests were carried out using UMT-5 (Bruker Nano Inc. Germany) under rotation modes in RO water against 5 mm diametric SiN balls with parameters of 50 mm min^−1^ speed, 0.5 N normal load, and 3 mm rotation diameter.

### 2.2 Cell experiments blood compatibility and in vitro protein absorption tests

NIH 3T3 cells needed for our experiment are the 3T3-L1 cell line from the cell bank of the Chinese Academy of Sciences and the primary fibroblasts were derived from PromoCell's NHDF-c product. Cells were cultivated in RPMI 1640 medium (Gibco) supplemented with 10 % heat-inactivated fetal bovine serum (Gibco) at 37 °C and 5 % CO_2_. CCK-8 kits, Live-Dead staining kits and all the other biochemicals were obtained from Beijing Solarbio Company. Detailly, for cytotoxicity evaluation [29], cells with a density of 1 × 10^4^ were seeded onto the samples. Then ILH with different concentrations (1 % and 2 %) of BAC crosslinkers were extracted. The cells were cultured in a medium with a 1:10 (v/v) ratio of ILH to normal medium, and 100 μL of extract was added to each well. For CCK8 tests, the pore plates were removed from the cell incubators at different time points (1 and 3 days), 10 μL CCK-8 solution was added to the cell incubators, and incubated in the cell incubators for 2 h under dark conditions. Finally, the data were read and recorded with an enzyme-labeled instrument (wavelength selected as 450 nm). For Live/Dead staining, we prepared the live/dead working fluid by adding 4 μL of CalcIN-AM and 6 μL of PI to 2 mL of PBS solution under full light protection conditions, and thoroughly mixed it with a pipette. Then 200 μL of the prepared dye was added into each hole of the 96-well plate and incubated at 37 °C for 30min. Finally, the excess stain was cleaned with PBS, and 50 μL common cell medium was added. Finally, they were observed and photographed under a confocal fluorescence microscope (Leica, Germany).

During the blood compatibility test, the rat tail tip was cut off, and blood could be seen flowing out of the tail tip. 5 mL of SD rat tail vein blood was collected by vacuum blood collection vessel containing anticoagulant with scissors, and red blood cells were separated from the blood by centrifugation. 0.5 mL ILH and 0.5 mL erythrocyte stock solution were mixed into 1 mL EP tube and then shaken for 1 h 0.9 % NaCl and TritonX-100 (0.1 %) were used as negative control and positive control, respectively. Then the centrifuged 0.1 mL supernatant was placed into the 96-well orifice plate. Finally, the hemolysis rate (%) = [(Ap -- Ab)/(At -- Ab)] × 100 was determined by enzyme-labeled instrument (absorbance at 540 nm). Among them, Ap, At and Ab represent the absorbance of sample group, TritonX-100 group and 0.9 % NaCl group at 540 nm, respectively.

The standard BCA method was used to evaluate the adsorption of non-specific proteins on discs and ILH. Firstly, cut ILH into discs with 12 mm diameter and 0.2 mm thickness, and then place them into a 48 well cell plate. 400 μ L BSA PBS solution (1 mg/mL) was added to each well, and then incubated at 37 °C. After 2 h, aspirate the protein soaking solution and wash the well repeatedly with PBS. Subsequently, add 400 μL of sodium dodecyl sulfate solution (SDS, 1.0 wt%) to each well and slowly shake well for 1 h. The protein concentration in the solution was determined by the BCA assay method. An enzyme-linked immunosorbent assay (Bio Tex Epoch, Bio Tex, USA) was used to measure the absorbance value at 562 nm and the content of BSA in the corresponding eluent was calculated based on the measured BSA standard curve.

### 2.3 Animal experiments

All the animal experiments were approved by Hebei University Animal Ethics Committee (Support No. 202405). Thirty SD male rats were separated into three groups (n = 10 for each group): Model group, PEG group and ILH group. For model group, SD rat Achilles tendon postoperative adhesion model was established on 0 d without further action during the whole *in vivo* experiments. The detailed process was as following [30]: 5 % chloral hydrate anesthetic was injected into the rats' abdomen with a dosage of 10 mL/kg. After the rats were completely anesthetized, the hair was removed with a blade, and the rats were fixed on the operating table and disinfected. Then, the left hand pulled the right hind foot of the rat, and the right hand made a posterior midline incision at the right hind heel of the rat with a scalpel. In case of bleeding in the incision, a sterile cotton ball was used to press timely to stop bleeding. Exposed Achilles tendons were cut lengthwise, and sutured with a 3-0 suture using the modified Kessler method at the midpoint of the muscle tendon transition and calcaneus insertion. 0.3 mL normal saline was injected to set as the control group. For ILH experimental group and PEG control group, 0.3 mL 1 % ILH was injected into the tendon suture surface, while 0.3 mL PEG (20 % w/v) solution was injected into the control group. Finally, the incision was closed with a 6-0 suture and sterilized. The rats were fed to keep the environment clean and prevent postoperative infection. Tendon tissues were all harvested on 14 d and 28 d, and used to evaluate adhesion score (Score 0 indicates that the adhesion area is almost non-adhesion. Score 1 indicates that the fibrillated adhesion is easy to be bluntly separated. Score 2 indicates that blunt separation is difficult; Score 3 indicates that the fibrillated adhesion cannot be bluntly separated; Score 4 means that the adhesion is hard to be separated by sharp anatomical tools; Score 5 represents that the adhesion area must be separated with a sharp dissecting tool.), H&E and Masson staining. Immunofluorescence of TNF-α, IL-1, IL-6 and TGF-β1 were performed on 14 d, and p-smad2 and p-smad3 were performed on tissues taken on 28 d. RNA was extracted by Trizol (Invitrogen, USA) and analyzed by qPCR for collagen type I alpha 1 (COL1A1) and collagen type I alpha 1 (COL3A1). The primers for COL1A1 are 5′-CCCAGCGGTGGTTATGACTT-3' (sense), 5′-TCGATCCAGTACTCTCCGCT-3' (antisense). And the primers for COL3A1 are 5′-GTCGGAGGAATGGGTGGCTAT-3' (sense), 5′-CATTGCGTCCATCAAAGCCTC-3′(antisense). At the same time, tensile properties of the repaired tendons were measured by universal tensile tester.

### 2.4 Statistical analysis

Quantitative data were expressed as mean ± standard deviation, and the Shapiro-Wilk test was used to verify the normality of the data. When the data were normally distributed, for both sets of data, a two-sided paired *t*-test was used to determine their statistical significance. For the three data sets, a one-way ANOVA was used to determine their statistical significance. Statistical analysis was performed using SPSS statistical software (version 19.0). Statistical significance was set to ∗ P < 0.05, ∗ ∗P < 0.005, ∗∗∗P < 0.001.

## 3 Results and discussion

### 3.1 Verification on successful preparation and physical-chemical property of ILH

As described above, ILH was prepared based on free radical polymerization. Inserted SEM images from Fig. 2a show that ILH still possesses unique hydrogel morphology after repeatedly suction and extrusion, indicating that ILH was successfully prepared with shear-responsive injectable property. It is worth mentioning that the hydration-lubricative zwitterionic PSBMA within ILH facilitates water adsorption during gel formation. The concentration of crosslinker BAC affects the obtained ILH's gel speed, therefore samples with 0.1 %–3 % w/w BAC were evaluated for gel formation within 3 days, as displayed in Fig. 2b. After 1 d, 1%BAC group has been in gel appearance, as well as 2%BAC and 3%BAC groups. However, 0.1%BAC group cannot form full gel even on 3 d. To further confirm the minimum concentration for fastest gel formation, 0.5%BAC and 0.8%BAC groups were also compared. The results show that both 0.5%BAC and 0.8%BAC samples took at least 2 days to form gel state, while 1%BAC only needed 1 day. These phenomena illustrate that 1 % may be the optimal minimum crosslinker BAC concentration during material preparation. Fig. 2c show that ILH is widely injectable through different microneedles with varied sizes from 18G to 22G, which meets almost all clinic surgical requirements. ILH is also ease of storage and use, as Fig. 2d clearly displayed. ILH can be easily freeze-dried, transported and water-recovered until clinic use, which determines ILH may be very suitable as clinic products.

**Fig. 3:**
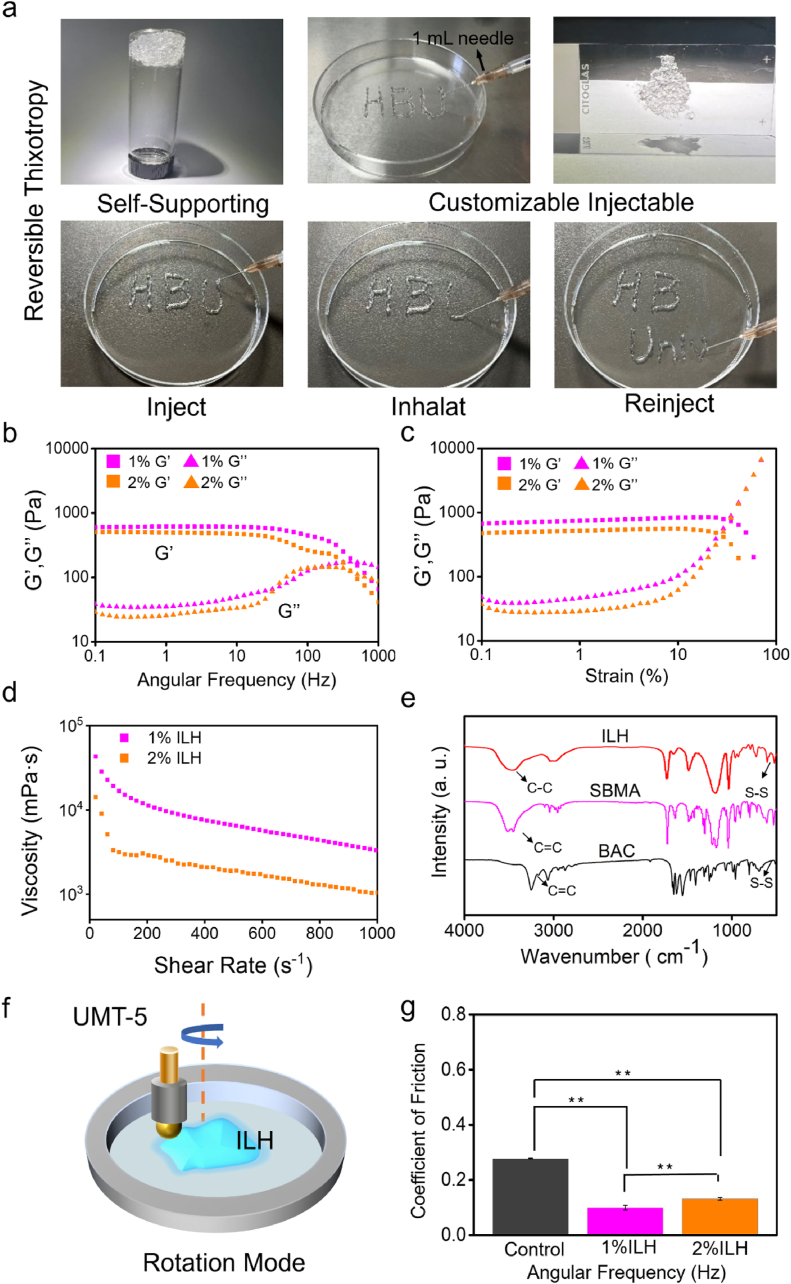
Physical performance characterizations of ILH. (a) Photos of evidence upon reversible thixotropy property. (b) Frequency-dependent (under 1 % strain, 25 °C) and (c) strain-dependent (at 10 rad/s frequency, 25 °C) oscillatory sweeps of cream gel formulations with different crosslinker content (1 or 2 % BAC). (d) Determination on shear-thinning property based on rheological tests. (e) Infrared spectrum of BAC, SBMA and ILH. (f) Schematic diagram of friction measurement. (g) Comparison of COF value between 1%ILH and 2%ILH samples based on the tribological test operated under a rotation mode (n = 5 independent experiments, ∗*P* < 0.05, ∗∗*P* < 0.005).

The developed ILH possess reversible thixotropy based on its dynamic disulfide bonds. As shown in Fig. 3a, ILH can be self-supported and adhered onto an up-side-down glass container bottom after normal injection. This indirectly indicates that ILH can protect tissue under no motion stimuli. In addition, it can be seen from Fig. 3a that ILH is not only customized injectable but also reversible shear-responsive, which is achieved by dynamic disulfide bonds within stable hydrogel network. FTIR data from Fig. 3e distinctly show the disulfide bonds within ILH, which confirm once again the reversible sol-gel formation mechanism as discussed in *Introduction* part. Moreover, to further study rheological property of ILH, storage modulus (G′) and loss modulus (G″) under oscillation modes were conducted via a rheological instrument. Fig. 3b and c displays curve data with abscissa of angular frequency and abscissa of strain, respectively. When angular frequency varied from 0.1 % to 1000 % with strain remained still (Fig. 3b), 1%-ILH and 2 % -ILH samples both reveal an increasing trend in G″ and a decreasing trend in G′, and meanwhile G′ stay higher than G’’. This indicates ILH remained gel form during the whole tests. Besides, when strain varied from 0.1 % to 100 % with angular frequency stayed constant (Fig. 3c), especially higher than 10 % which corresponds to human soft tissue strain [31,32], it can be witnessed that ILH samples both have a clear increase trend in G″ and a clear decrease trend in G’. These data illustrated that ILH may protect tendon surface as gel appearance under continuous shearing. To further prove the shear-thinning property of ILH, data curve of shear rate – viscosity was also collected and is shown in Fig. 3d. 1%ILH and 2%ILH samples both showed an obvious decrease on viscosity when shear rate increases, which is the typical feature for shear-thinning. Therefore, the shear-responsive performance of ILH has been determined. To confirm the lubricative performance of ILH, friction tests with rotation mode were carried out (Fig. 3f) and the data is shown in Fig. 3g. The coefficient of friction (COF) data show that ILH samples possess significantly lower COF value (∼0.1) than that of control group (∼0.25), with more than two-fold reduction. All these data definitely prove ILH is motion-responsive and lubricative.

### 3.2 *In vitro* bio experiments upon cytocompatibility and protein-resistance performance of ILH

**Fig. 4:**
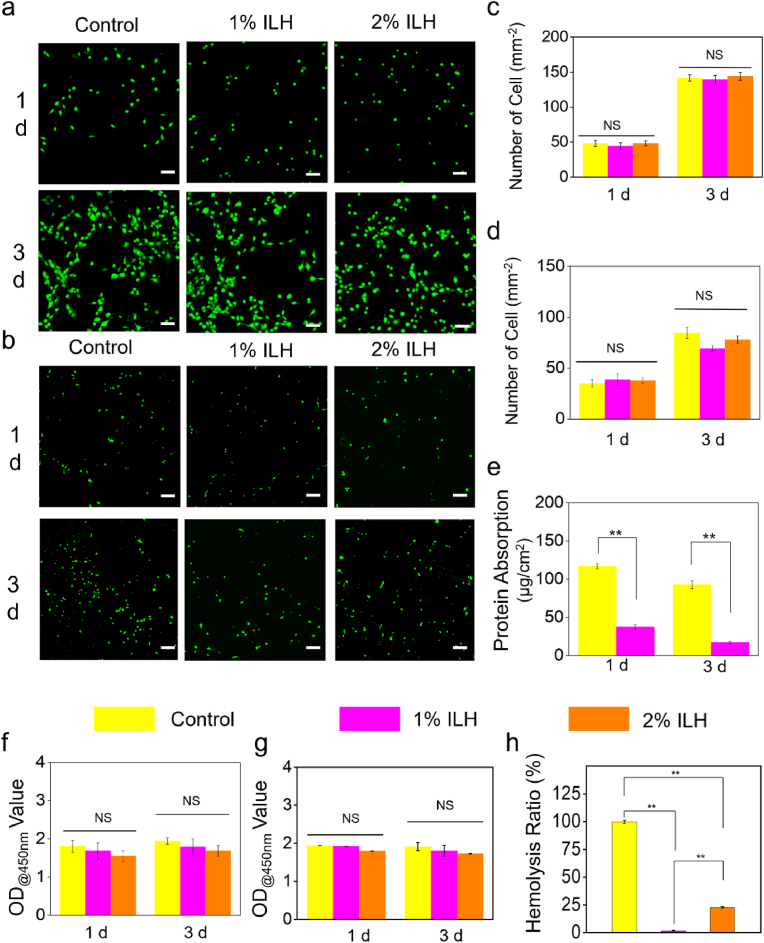
*In vitro* cytocompatibility and anti-adhesive tests. Live/dead staining of (a) NIH/3T3 cells and (b) primary fibroblasts after incubated with the ILH extract for 1 and 3 days. Scales bar: 100um. Density of (c) NIH/3T3 cells and (d) primary fibroblasts on 1 and 3 days. (e) Protein absorption. CCK-8 test data of (f) primary fibroblasts and (g) NIH/3T3 cells. (h) Hemolytic percentage. ∗∗*P* < 0.005. NS: no significance.

To evaluate the cytocompatibility of ILH, NIH 3T3 fibroblasts from continuous cell lines and primary cells were both chosen. Firstly, live/dead staining images on 1 d and 3 d for both cell lines were recorded to observe cell growth condition after co-cultured with ILH. It can be clearly seen from Fig. 4a and b that there are no obvious differences upon cell growth between sample groups, where no red-stained dead cells were found, qualitatively indicating their optimal cytocompatibility. Semi-quantitative data in Fig. 4c and d is also in accord with this phenomenon, verifying again the optimal cell compatibility of the developed ILH. In addition, CCK-8 measurements were conducted and the results are shown in Fig. 4f and g. OD values at 450 nm of each group do not have statistical differences, meaning that 1%ILH and 2%ILH samples both did not have cytotoxicity. Therefore, based on the qualitative and semi-quantitative data in vitro, it can be certainly demonstrated that ILH possesses proper cytocompatibility.

Blood compatibility is another important aspect for biomaterials' biocompatibility. Therefore, we tested ILH's blood compatibility in vitro and the related data is shown in Fig. 4e. Both 1%ILH and 2%ILH groups have significantly lower hemolysis ratio (<25 %) than control group, which determines ILH's proper blood compatibility. Considering 1%ILH group possesses lower hemolysis ratio than that of 2%ILH group, 1%ILH is chosen for the following animal experiments. Tendon adhesion is connected to protein absorption; hence protein absorption tests in vitro were carried out. The corresponding data displayed in Fig. 4h clearly indicates ILH possesses significant anti-protein-absorption performance, which is important for *in vivo* adhesion prevention [33,34].

### 3.3 *In vivo* biofunction proof of ILH

**Fig. 5:**
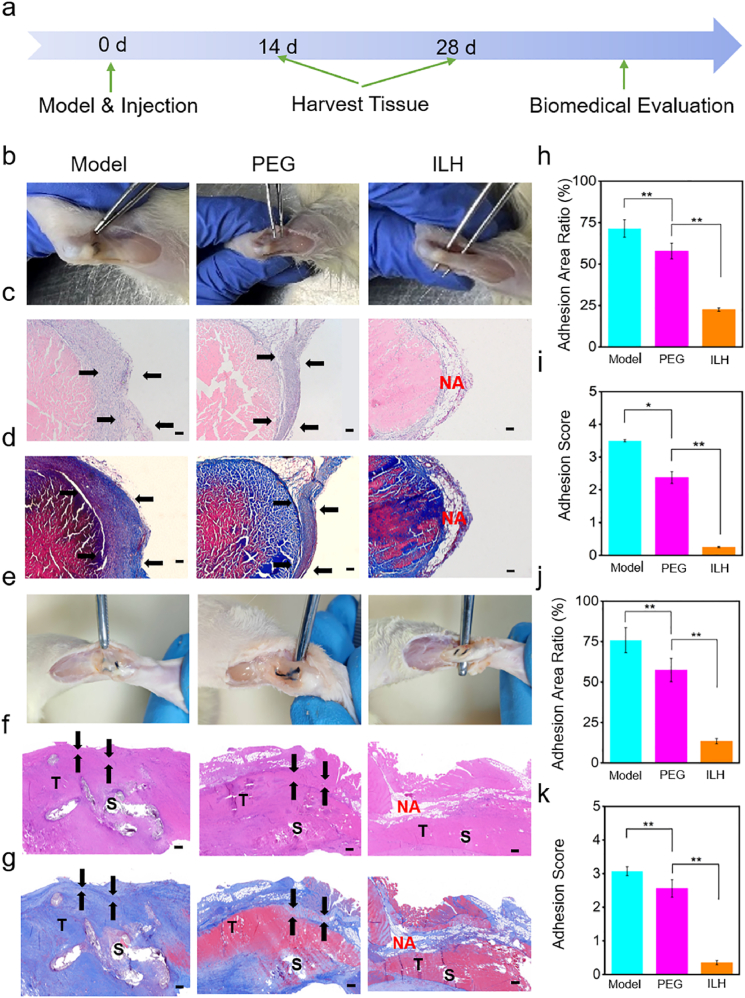
*In vivo* biofunction evaluation of ILH on rat tendon adhesion model. (a) Schematic diagram of the overall animal test process. (b, e) Photos, (c, f) H&E and (d, g) Masson staining images of the harvested tendon on 14 d and 28 d after implantation, respectively. Scale bars: 200 μm. Black arrows point to adhesion sites. NA: No adhesion. S: Suture sites. T: Tendon. (h, j) Adhesion area ratios and (i, k) adhesion scores on 14 d and 28 d, respectively. ∗*P* < 0.05, ∗∗*P* < 0.005.

As shown in Fig. 5a, SD rat Achilles tendon postoperative adhesion evaluation on 14d and 28d is done for *in vivo* biofunction proof of the developed ILH. PEG is a representative hydrophilic polymer that cannot form strong hydration layer as zwitterionic polymer does. As we above discussed, ILH that containing zwitterionic pSBMA is expected to locally protect tendon when rest, and be lubricative when tendon slides. This specific performance would provide an ideal condition for non-adhesive high quality tendon repair. Also, PEG cannot achieve shear-responsive performance as ILH does. To better clarify the proper hydration lubrication performance and motion-responsive property of ILH, therefore PEG was chosen as the control group in animal experiments. In practice, the collected *in vivo* data verify this expectation.

**Fig. 6:**
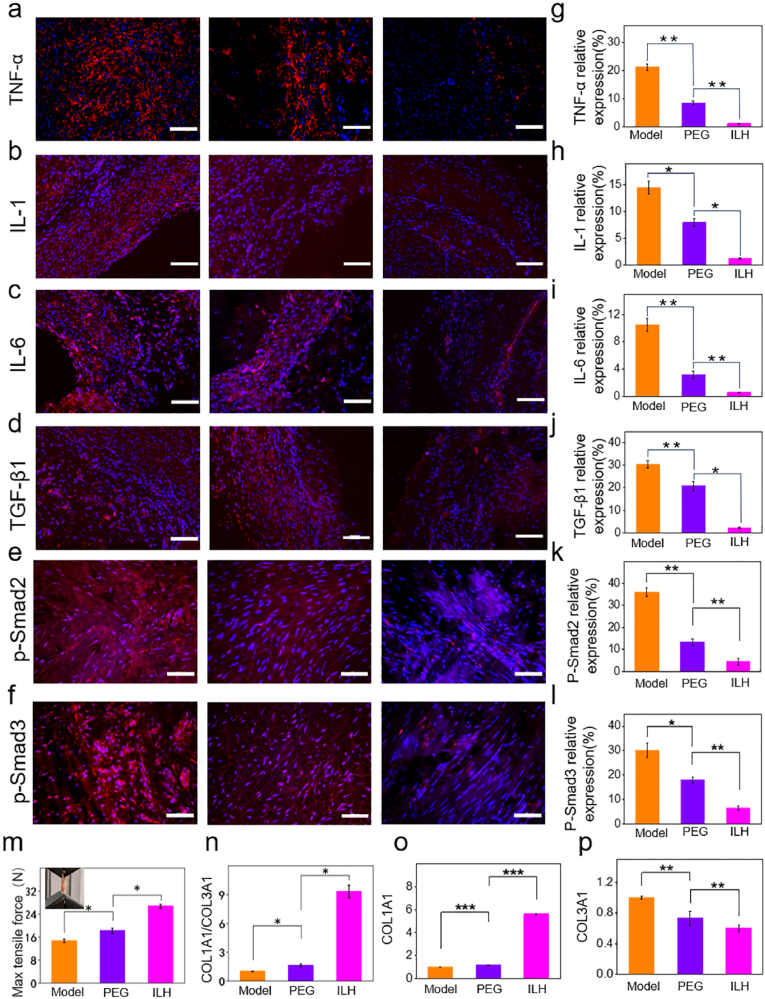
The immunofluorescence staining and corresponding semi-quantitative data of (a, g) TNF-α, (b, h) IL-1, (c, i) IL-6, (d, j) TGF-β1, (e, k) p-Smad2, (f, l) p-Smad3 in the model, PEG, and ILH groups (Red color represents targeted protein and blue color represents cell nucleus. scale bar:100 μm). (m) Max tensile forces of repaired tendon in each group. The Relative DNA expression of (n) COL1A1/COL3A1, (o) COL1A1 and (p) COL3A1 based on qPCR tests. ∗*P* < 0.05, ∗∗*P* < 0.005, ∗∗∗*P* < 0.001. (For interpretation of the references to color in this figure legend, the reader is referred to the Web version of this article.)

14 days after surgery, surgical site skin on the right hind limb of the rat was opened, and the Achilles tendon was exposed for photography and data recording, as shown in Fig. 5b. It can be clearly seen from the collected tissue photos that there is no obvious tissue inflammation or ulcer in the surgical limbs of each group. Surgical forceps in ILH group can easily pass through the area between the tendon and the underlying fibrous tissue, but it is difficult for the other control groups and PEG groups to pass through. This indicates that the control group and PEG group had severe Achilles tendon adhesion, while the ILH group had almost no adhesion. We evaluated the adhesion scores in each group based on the Achilles tendon adhesion score standard and the data is shown in Fig. 5i. According to the adhesion scoring criteria, we need to intervene with round headed forceps (blunt) and surgical scissors (sharp) to separate the adhesive tissue and speculate on the specific score. It is shown that the Model group has a significant highest adhesion score (score 3–4), predicting severe adhesion at the tendon fiber tissue interface. In addition, the degree of adhesion between the Achilles tendon and surrounding tissues in the ILH group (score <1) was significantly lower compared to the PEG control group (score 2–3), indicating that ILH has better anti adhesion performance compared to PEG. As shown in Fig. 5c, we used H&E staining to observe tissue adhesion at cellular scale. By zooming the surrounding tissues of the Achilles tendon, it was evident that the control group has severe fibrosis and a large amount of inflammatory cell infiltration. The PEG group also has severe adhesion and a large amount of inflammatory cell infiltration, while the control group has significantly smaller adhesion and inflammatory cell infiltration around the Achilles tendon compared to the control group and PEG group. We also used Masson staining results to visually understand the amount of collagen fibers around the Achilles tendon (Fig. 5d), in order to identify the adhesive strength of the tissue. It can be observed that there are many collagen fibers adhering around the Achilles tendon in the control group and PEG group (blue in color), and severe and excessive fibrosis can be clearly observed in the control group and PEG group, while there is little collagen fiber adhesion around the Achilles tendon in the ILH group. This indicates that the control group and PEG group has more severe Achilles tendon adhesion, while the ILH group has almost no Achilles tendon adhesion. In order to further evaluate the degree of rat Achilles tendon adhesion between different groups, we combined H&E staining and Masson staining to statistically calculate the adhesion area of each stained image (Fig. 5h). It can be found that model group showed adhesion area around 75 %, indicating that the rats in the model group were more prone to larger areas of adhesion and severe adhesion. PEG group possessed adhesion area greater than 50 %, indicating that the PEG group do not have a significant preventive effect on postoperative adhesion. It is worth noting that the adhesion area in ILH group showed a smallest value (less than 25 %), indicating that the implantation of ILH *in vivo* after surgery has a significant anti-tissue-adhesion effect. An extended 28-day rat tendon adhesion evaluation experiments were also conducted to further determine ILH's anti-adhesive performance and explore the mechanism in molecular level. The time point of 28 days was referenced to previous literature [35,36]. It can be seen from the photos (Fig. 6e) that only ILH group's tendon was easy for tweezer to pass, and it possessed significantly lowest adhesion scores with only about 0.3 that was one-tenth of model group and one-eighth of PEG group (Fig. 6k). This result preliminarily indicates ILH possesses significant anti-adhesive property in a relative long-term rat tendon adhesion model. H&E and Masson staining photos from Fig. 6f and g pathologically support this conclusion. ILH group showed no adhesion and little inflammation, while the other groups showed clear adhesion and severe inflammation. The corresponding adhesion area ratio data analyzed from histological staining (Fig. 6j) also prove this phenomenon.

**Fig. 7:**
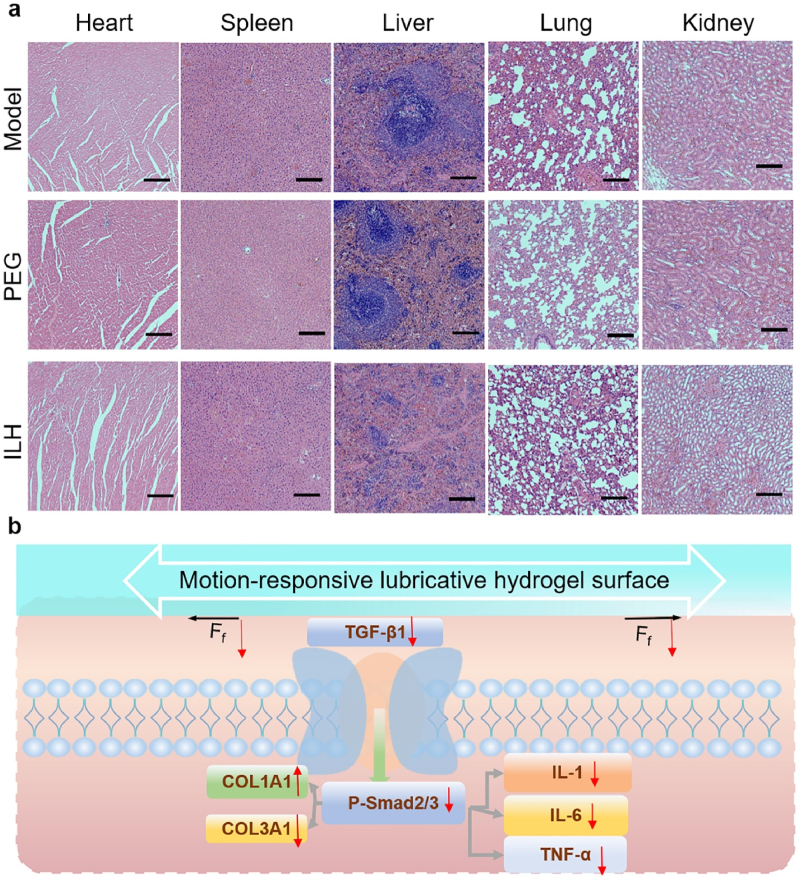
(a) H&E staining photos of heart, liver, spleen, lung and kidney on 28 days' animal experiments. (b) Schematic illustration for anti-adhesion mechanism in molecular level.

Inflammation is an important factor leading to tissue adhesion, which can exacerbate fibrosis and adhesion formation [[37], [38], [39], [40]]; hence we also assessed expression tests of TGF-β1-p-Smad2/3 signal pathway related proteins (TNF-α, IL-1, IL-6, TGF-β1, and p-Smad2/3) and genes (COL1A1 and COL3A1) by immunofluorescence staining (Fig. 6a-l) and qPCR (Fig. 6n-p). As expected, the fluorescence microscope scanning images (Fig. 6a-f) and corresponding semi-quantitative results (Fig. 6g-l) showed that the expression levels of TNF - α, IL-1, IL-6, and TGF - β1 as well as p-Smad2/3 in the ILH group were significantly lower in statistical than those in the other two groups. This undoubtedly indicates that ILH can reduce inflammatory response, block the adhesion of inflammatory cells and cytokines in the surrounding tissue of the rat Achilles tendon, and significantly prevent tendon adhesion. Based on the data displayed from Fig. 6n to p, it can be witnessed that the gene expressions of COL1A1/COL3A1 were significantly highest in ILH group due to its significantly highest COL1A1 gene expression and lowest COL3 expression. This result indicates that ILH further led to the healing promotion rather than adhesion formation. These results all demonstrate our developed ILH could promote high-quality tendon repair without adhesion. Tensile properties of repaired tendon for each group were conducted, as shown in Fig. 6m. ILH group had significantly highest tensile strength than model group and PEG group, with an max tensile force around 25 N. To further confirm ILH's biosafety *in vivo*, the rat visceral organs including heart, liver, spleen, lung and kidney were harvested and H&E stained. The results are displayed in Fig. 7a and clearly show that ILH did no harm to rat visceral organs which means proper *in vivo* biosafety.

TGF-β1-p-Smad2/3 signal pathway [41,42], which is activated by friction, is very important for proving lubricative biomaterials’ mechanism. The Smad protein family plays an important role in TGF-β1 signal transduction [41]. By binding TGF-β1 to cell surface receptors, activator Smads (Smad2/3) are recruited and then phosphorylated, and the phosphorylated Smad are then translocated to the nucleus, where they function as transcription factors to adjust target genes. Type I collagen is the most abundant component in the extracellular matrix of tendon tissue, and the proportion of type III collagen in tendon tissue increases during tendon scar repair [42]. As shown in Fig. 7b, ILH possesses motion-responsive performance, being very lubricative when tendon slides, which significantly reduces friction and thus further inhibits the activation of TGF-β1-p-Smad2/3 signal pathway. Due to this mechanism, high quality tendon repair without adhesion is realized.

Overall, the *in vivo* experiments’ data have demonstrated the excellent performance of our developed ILH upon Achilles tendon adhesion prevention. To date, the clinic-used injectable products for preventing tendon adhesion are hydrophilic polymers, such as hyaluronic acid [43]. Although lubricative hyaluronic acid has been proven to be effective to a degree, it cannot adapt to the real complex process during tendon repair. As well known, Achilles tendon is a motor organ that ensures the sliding of joint and bones. Therefore, the ideal product for high-quality tendon repair should be smart and dynamic, being motion-responsive: be highly lubricative at fluid state when tendon moves, while be less lubricative at solid state to protect tissue endogenous healing when tendon rest. Compared with hyaluronic acid, our developed ILH not only possessed better lubricating property based on hydration lubrication theory, but also possessed motion-responsive performance that is suitable for real clinic use.

## 4 Conclusion

In this work, we developed a motion-responsive injectable lubricative hydrogel (ILH) based on dynamic disulfide bond and hydration lubrication theory. ILH is proven to be successfully prepared with special shear-responsive thixotropy, thus being customized injectable. Also, ILH is ease of storage and reuse, which just needs frozen dried and water recovery. ILH has proper biocompatibility, strong protein resistance and anti-cell-adhesion performance. The data of rat tendon postoperative adhesion model evaluation experiments on 14 d and 28 d both determined that ILH facilitates tendon non-adhesive healing due to its specific motion-responsive lubricative ability. It can be supposed that besides for application of tendon high-quality repair, ILH is an ideal product for other complex postoperative surgical repair issues, for example friction-induced osteoarthritis. We believe the special design in this work would open a novel horizon for injectable biomaterials developments.

## CRediT authorship contribution statement

**Shujie Cheng:** Resources, Funding acquisition, Data curation. **Jihong Yang:** Investigation. **Jianguo Song:** Data curation. **Xin Cao:** Data curation. **Bowen Zhou:** Data curation. **Lan Yang:** Writing – original draft. **Chong Li:** Funding acquisition. **Yi Wang:** Writing – review & editing, Writing – original draft, Supervision, Resources, Project administration, Methodology, Investigation, Funding acquisition.

## Declaration of competing interest

All authors disclosed no relevant relationships.

## Data Availability

Data will be made available on request.
